# Data on the effects of anti-cancer drug of resveratrol in breast cancer cells, MDA-MB-231 cells

**DOI:** 10.1016/j.dib.2017.03.029

**Published:** 2017-03-21

**Authors:** Eunmi Park

**Affiliations:** Department of Food and Nutrition, School of Life Science and Nano-Technology, Hannam University, Daejeon, South Korea

**Keywords:** Resveratrol, Chemotherapy, Natural bioactive compounds

## Abstract

The data here is related to the article, “Curcumin enhances poly (ADP-ribose) polymerase inhibitor sensitivity to chemotherapy in breast cancer cells” (Y.E Choi, and E. Park, 2015) [1]. The article shows that curcumin, as a natural bioactive compound, enhanced DNA damage response and induced cell death in MDA-MB-231 cells [1]. This data includes that breast cancer cells, MDA-MB-231 respond to DNA damage after UV irradiation, post to resveratrol treatment. The data shows that resveratrol treatment results in reduction of S-phase cell cycle and induction of γ-H2AX, which is a hallmark of DNA damage after UV irradiation in breast cancer cells, MDA-MB-231. Moreover, resveratrol sensitizes breast cancer cells to respond to UV treatment as a natural bioactive compound.

**Specifications Table**

**Table:** 

Subject area	Biology
More specific subject area	Cancer biology
Type of data	Figure, graph
How data was acquired	FACS analysis, colony assay and western blot
Data format	Analyzed
Experimental factors	Comparison of resveratrol with UVC irradiation in MDA-MB-231 cells
Experimental features	Cell cycle analysis, survival data and western blotting in breast cancer cell line, MDA-MB-231 with resveratrol treatment post to UVC irradiation
Data source location	Daejeon, Korea
Data accessibility	Data is within this article

**Value of the data**

• Dana can be used for investigate the cell cycle effects of resveratrol on UVC irradiation.
• The data provides the information of the synergistic effect of resveratrol in combination of the UVC irradiation in breast cancer cell culture system.
• Data will be useful for investigating the effect of resveratrol on DNA damage response in breast cancer cells.
• This data significantly extends the effects of resveratrol in breast chemotherapy.

## 1 Data

**Fig. 1:**
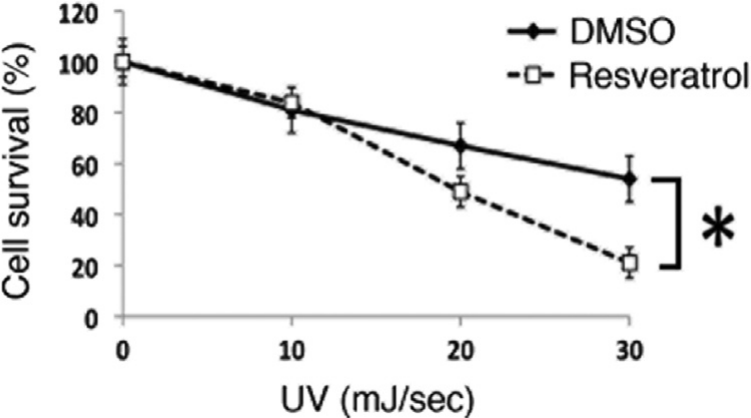
Resveratrol sensitizes MDA-MB-231 breast cancer cells to UVC treatment. MDA-MB-231 cells were pretreated with resveratrol (10 μM) or DMSO for 24 h and re-plated in culture dishes. Then the cells were exposed with various UVC irradiations (0–30 mJ/sec). Survival was determined using a colony assay from three independent experiments. The data are mean±standard errors. *; *p*<0.05.

**Fig. 2:**
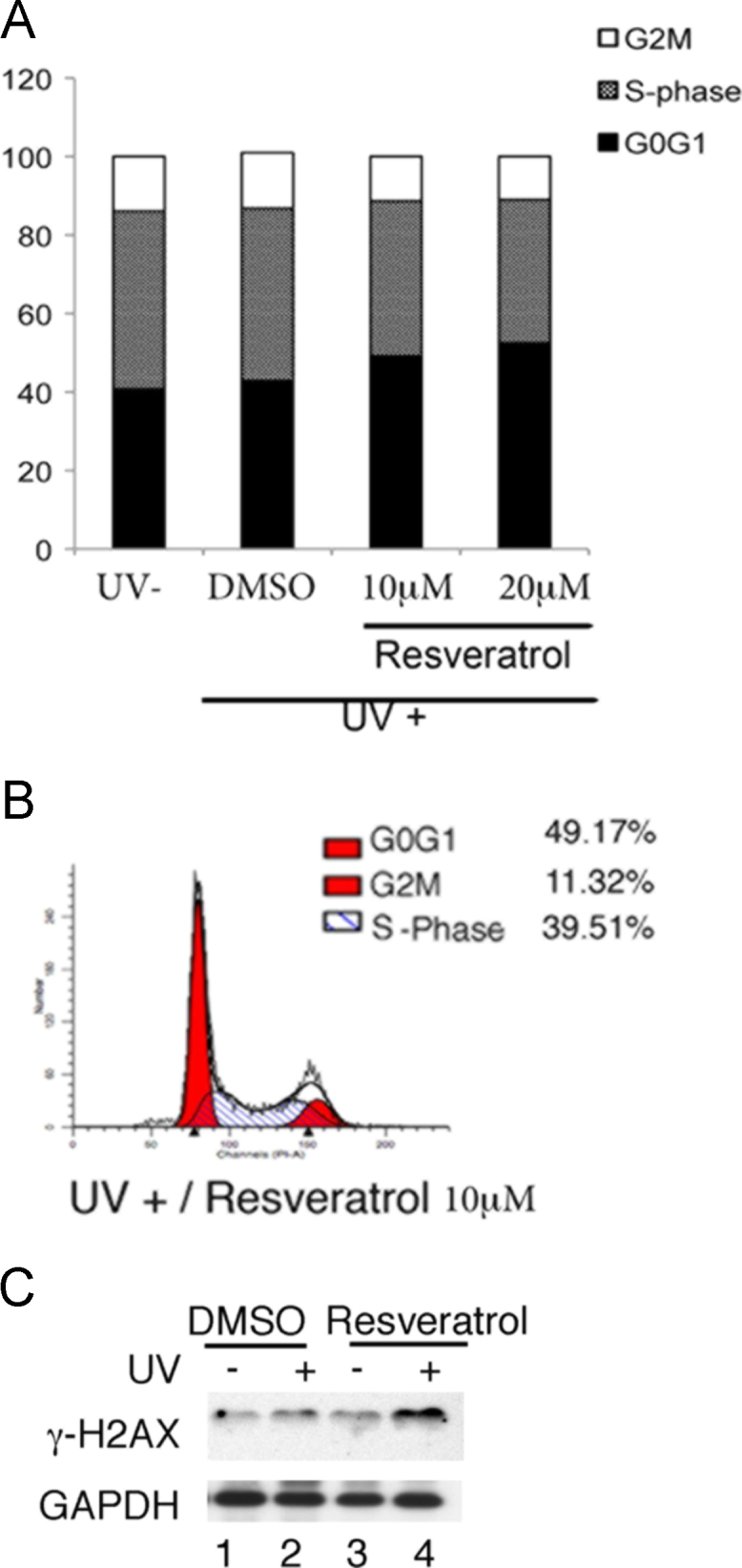
Resveratrol shows the reduction of S-phase cell cycle profiles post to UVC treatment. (A) MDA-MB-231 cells were cultured with 10 μM, 20 μM resveratrol/or DMSO for 24 h. The cells were exposed to UVC treatment and harvested. The cell pellets were fixed in 70% ethanol and stained with PI for FACS analysis. (B) Representative data of FACS analysis data in MDA-MB-231 cells with 10 μM resveratrol treatment post to UVC treatment. (C) Western blotting in MDA–MB-231 cells with 10 μM resveratrol treatment post to UVC treatment. UV−; UV untreated, UV+; UV treated (30 mJ/sec, harvest post to 3 h).

Resveratrol hyposensitized breast cancer cells to various UVC irradiations (0–30 mJ/sec) compared to cells treated with DMSO (Fig. 1). Next, resveratrol reduced more the S-phase cell cycle profiles post to 30 mJ/sec of UVC-induced DNA damage, compared to the cells treated with UVC alone (See Fig. 2A and B).

In addition, the effect of resveratrol on response of γ-H2AX induced by UVC treatment was provided in the western blotting (Fig. 2C).

## 2 Experimental design, materials and methods

### 2.1 Cell culture

Breast cancer cell line, MDA-MB-231 cells were cultured in Dulbecco׳s modified Eagle׳s medium (DMEM) (Invitrogen) containing 1% penicillin/streptomycin and 10% fetal bovine serum (Invitrogen) [1], [2], [3]. Resveratrol (Sigma) was dissolved in DMSO. The MDA-MD-231 cells were treated with resveratrol for all of experiments. The cells were incubated at 37 °C with 5% CO_2_.

### 2.2 Cell cycle analysis

For fluorescence-activated cell sorting (FACS) analysis, MDA-MB-231 cells were fixed overnight at 4 °C in 70% ethanol, stained with propidium iodine (PI) for 1 h. The cells analyzed for DNA content using a FACS Calibur machine (BD Biosciences) [4].

### 2.3 Colony assay (Cell survival analysis)

Cell survival analysis was performed as described previously [3], [5], [6]. MDA-MB-231 breast cancer cells were prepared and exposed to resveratrol or DMSO for 12 h. After the treatment with UV irradiation (0–30 mJ/sec), the cells were re-plated in 6-well plates for clonogenic assays in triplicate. After 2 weeks later, cell culture were stopped and fixed with methanol. The colonies were stained with crystal violet. Data are mean±SEM as indicated. Statistical significance of comparison between two groups was determined by two-tailed Student׳s The *p*-values of less than 0.05 considered significant differences of statistical analysis.

### 2.4 Western blot

Cell lysates (30 μg) was applied to 10% SDS-PAGE gel, and then transferred to nitrocellulose membranes. Specific protein levels were measured by Western blotting as described previously [1], [2], [3] with the antibodies against γH2AX (Cell signaling) and GAPDH (Bethyl laboratory).
